# Lateral Proboscis (Elephant Tusk) with Orofacial Clefts: A Report of a Rare Case

**DOI:** 10.1155/2018/6820972

**Published:** 2018-09-27

**Authors:** Sunday O. Ajike, Rabiu Mohammed, Adegbenga O. Johnson, Kato Chaha, Modupe O. Samaila

**Affiliations:** ^1^Maxillofacial Unit, Ahmadu Bello University Teaching Hospital, Zaria, Kaduna State, Nigeria; ^2^Anaesthesia Department, Ahmadu Bello University Teaching Hospital, Zaria, Kaduna State, Nigeria; ^3^Chaha Eye Hospital, 42B Jabi Road, Kaduna, Kaduna State, Nigeria; ^4^Department of Histopathology, Ahmadu Bello University Teaching Hospital, Zaria, Kaduna State, Nigeria

## Abstract

Lateral proboscis is a rare congenital anomaly. Lateral proboscis is a rare craniofacial malformation characterized by a rudimentary tubular, nose-like structure occurring in association with a wide spectrum of other anomalies. We presented a seven-month-old girl's lateral proboscis, cleft lip, and palate. Proboscis was excised by an elliptical incision, and the cleft was repaired at the same surgery.

## 1. Introduction

Proboscis lateralis is a very rare congenital anomaly characterized by a rudimentary tubular, nose-like structure arising from the medial portion of the orbital roof [1] with an incidence of 0.1 : 10,000 [2]. It is frequently associated with other abnormalities: heminasal aplasia/hypoplasia, mental retardation, callosal agenesis, encephalocele, microphthalmia, holoprosencephaly, and clefts [3–6], or may occur in isolation [4, 5]. It may be unilateral or bilateral [3, 5, 6] with a 2 : 1 male-female preponderance [5]. According to Guerrero et al [1], it was first reported in 1861 by Forster in his monograph congenital malformations of the human body, while Boo-Chai [5] suggested a classification of this entity in 1985, and it was subsequently modified by Sakamoto et al. [7] in 2012.

Diagnosis is usually clinical and radiological [8–10]; however, the use of ultrasound during antenatal was reported by Kolluru and Coumary [6] and Eroğlu and Uysal [10]. Surgery is the mode of treatment.

To the best of our knowledge, no case of lateral proboscis has been reported in our environment. Furthermore, the extensive search of the English literature yielded case reports and series. We therefore report this rare anomaly occurring in association with clefts of the lip and palate.

## 2. Case Report

A baby girl aged seven months presented to our clinic, Etomie Oral and Maxillofacial Clinic, Kaduna, Kaduna State, Nigeria, with a thick tubular structure in the medial canthus of the left eye and cleft of the left upper lip, left alveolus, and primary and secondary palates from birth. The antenatal history revealed nine months of uneventful gestation with uncomplicated spontaneous vaginal delivery. There was no prenatal history of exposure to alcohol, ionizing radiation, or drugs and consanguinity. The mother denied any family history of congenital anomalies of any type.

**Figure 1:**
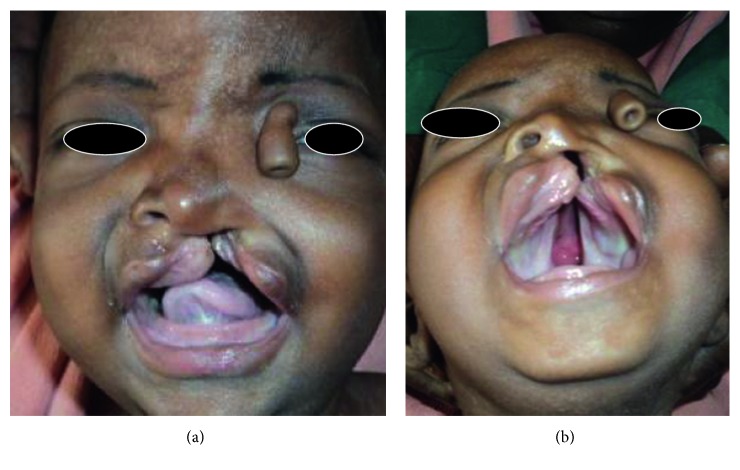
(a) Full face profile and (b) worm eye view showing lateral proboscis with complete clefts of the lip and the palate.

Clinical examination revealed an otherwise normal baby weighing 3 kg with a tubular fleshy structure measuring about 3 cm in the medial canthus of the left eye and cleft of the left upper lip, alveolus, and primary and secondary palates (Figure 1).

The globes and the nose were all normal. Other systemic examinations were also normal. Laboratory investigations were all within normal limits.

Based on this clinical presentation, a diagnosis of lateral proboscis with cleft of the left upper lip, alveolus, and palate was made, and the patient was subsequently prepared for surgery.

After routine cleaning and draping, the upper lip cleft was repaired using the straight-line technique, while the proboscis was excised using an elliptical incision.

**Figure 2:**
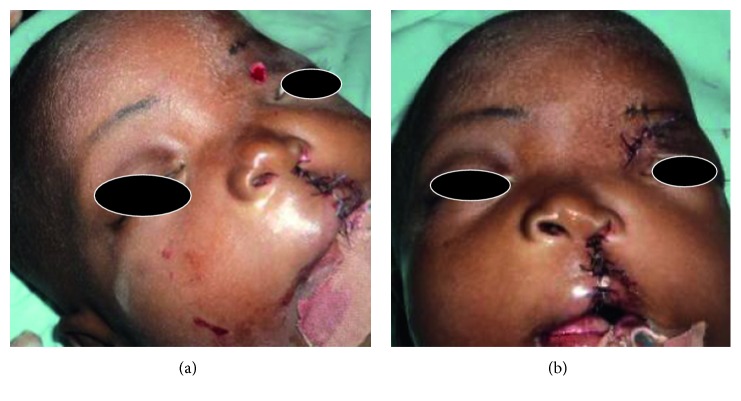
Surgical site (a) before and (b) after suturing.

The resultant wound was closed in layers using 4/0 Vicryl sutures (Figure 2).

**Figure 3:**
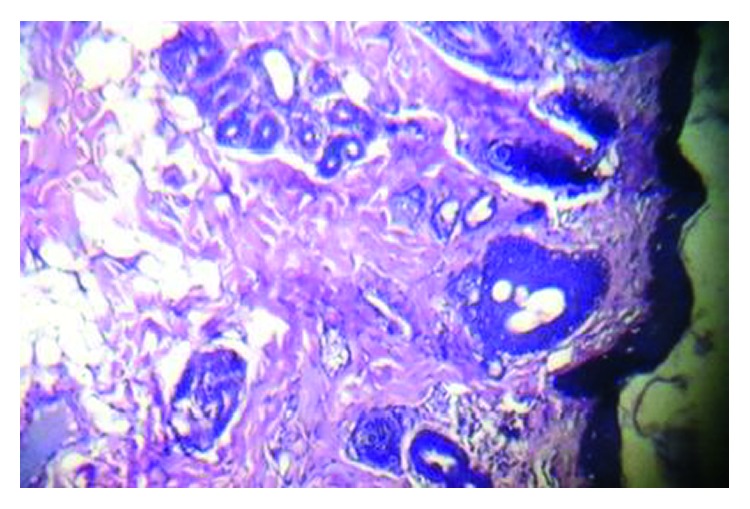
Photomicrograph showing the stratified squamous epithelium, overlying collagenized dermis containing adnexal structures, and admixture of fat lobules and collagen bundles (H&E × 10).

Gross examination of the specimen showed an oblong skin-covered tubular mass measuring about 3 cm, while histological examination showed the stratified squamous epithelium, overlying collagenized dermis containing adnexal structures, and admixture of fat lobules and collagen bundles (Figure 3).

The patient was managed with I.M. lincomycin 150 mg 8 hourly for 5 days, with I.M. pentazocine 7.5 mg statum, and thereafter with paracetamol 5 ml for 3 days. Sutures were removed 7 days postoperatively, and the patient was subsequently discharged the same day.

## 3. Discussion

Proboscis lateralis is a very rare congenital anomaly characterized by a rudimentary tubular, nose-like structure arising from the medial portion of the orbital roof [1]. It is usually in the medial canthus of the eye [3, 5]; it may be isolated [4] or in association with other craniofacial anomalies [3, 4, 6]. In our case, it occurred in association with another midline craniofacial anomaly: the cleft lip and palate (Figure 1). The cleft lip and palate defect occurs due to defect in the fusion of frontonasal, maxillary, and mandibular processes, while the proboscis lateralis occurs as a result of displacement of the nasal placode during the formation of the nose [1, 2]. The coexistence of the proboscis lateralis and the facial clefting in this case cannot be readily explained, except one speculates that this could be by freak of nature or that these two independent abnormalities are occurring concurrently during the gestation period of 4–6 weeks at which the frontonasomaxillary and mandibular processes are undergoing differentiation into the various organs. More specifically, since the nose, face, and palate usually develop at the same time during embryogenesis, we therefore hypothesized that there might have been an assault to these processes with resultant anomalies during embryogenesis.

The case presented here is a clinical diagnosis postnatally, whereas some authors [6, 11] have documented the use of ultrasound in the diagnosis of this condition, while CT and MRI evaluation helps to identify any cerebral anomalies [8, 9].

Proboscis lateralis usually occurs in association with absence of the nose on the same side [3, 5]; however, it may occur from an accessory nasal placode as seen in our case. Occurrence is usually in the midline; however, cases occurring in the nasal root [5], in the lateral canthus [5], bilaterally [6], and in the chin [12] have been documented.

Boo-Chai [2] suggested a classification of this entity proboscis lateralis in 1985 describing 4 groups depending on the clinical presentation:

  Group I: lateral proboscis with normal nose (least common).
  Group II: lateral proboscis with an ipsilateral deformity of the nose (second in frequency).
  Group III: lateral proboscis with ipsilateral deformity of the nose, eye, and/or ocular adnexa (the most common type).
  Group IV: lateral proboscis with ipsilateral deformity of the nose, eye, and/or ocular adnexa, plus the cleft lip and/or palate. However, Sakamoto et al. [7] proposed a new classification in 2012 following a review of 50 cases by adding 2 new groups to Boo-Chai's classification.
  Group V: lateral proboscis with encephalocele.
  Group VI: lateral proboscis with holoprosencephaly. The case reported here fits into group IV; except the eye and ocular adnexa, all were normal. We believe that the proboscis in this report was from the accessory nasal placode.

The surgical treatment of lateral proboscis depends on the clinical type. For group I, simple excision would suffice. In our case, simple excision (amputation) was done via elliptical incision since the noses were normal with satisfactory results (Figure 2) with repair of the cleft lip. For groups II–IV, a multistage and multidisciplinary approach is necessary. Furthermore, we suggest an early surgery to avoid psychological effects on the patients.

## 4. Conclusion

A rare case of lateral proboscis with clefts of the lip, alveolus, and primary and secondary palates treated by surgical excision and repair of the cleft lip is presented.
